# Use and awareness of and willingness to self-test for HIV: an analysis of cross-sectional population-based surveys in Malawi and Zimbabwe

**DOI:** 10.1186/s12889-020-08855-7

**Published:** 2020-05-25

**Authors:** Cheryl Johnson, Melissa Neuman, Peter MacPherson, Augustine Choko, Caitlin Quinn, Vincent J. Wong, Karin Hatzold, Rose Nyrienda, Getrude Ncube, Rachel Baggaley, Fern Terris-Prestholt, Elizabeth L. Corbett

**Affiliations:** 1 Global HIV, Hepatitis and STI programme, World Health Organization, Geneva, Switzerland; 2 Department of Clinical Research and Infection Disease, London School of Hygiene and Tropical Medicine, London, UK; 3 Department of Infectious Disease Epidemiology and MRC Tropical Epidemiology Group, London School of Hygiene and Tropical Medicine, London, UK; 4 Malawi-Liverpool Wellcome Trust, HIV/TB Group, Blantyre, Malawi; 5 Department of Clinical Sciences, Liverpool School of Tropical Medicine, Liverpool, UK; 6 U.S. Agency for International Development, Washington, DC USA; 7 Population Services International, Johannesburg, South Africa; 8 Ministry of Health, Lilongwe, Malawi; 9 Ministry of Health and Child Care, Harare, Zimbabwe; 10 Department of Public Health and Policy, London School of Hygiene and Tropical Medicine, London, UK

**Keywords:** HIV/AIDS, HIV self-test, HIV testing, Men, Sub-Saharan Africa, Population-based survey, Sexual risk behaviour

## Abstract

**Background:**

Many southern African countries are nearing the global goal of diagnosing 90% of people with HIV by 2020. In 2016, 84 and 86% of people with HIV knew their status in Malawi and Zimbabwe, respectively. However, gaps remain, particularly among men. We investigated awareness and use of, and willingness to self-test for HIV and explored sociodemographic associations before large-scale implementation.

**Methods:**

We pooled responses from two of the first cross-sectional Demographic and Health Surveys to include HIV self-testing (HIVST) questions in Malawi and Zimbabwe in 2015–16. We investigated sociodemographic factors and sexual risk behaviours associated with previously testing for HIV, and past use, awareness of, and future willingness to self-test using univariable and multivariable logistic regression, adjusting for the sample design and limiting analysis to participants with a completed questionnaire and valid HIV test result. We restricted analysis of willingness to self-test to Zimbabwean men, as women and Malawians were not systematically asked this question.

**Results:**

Of 31,385 individuals, 31.2% of men had never tested compared with 16.5% of women (*p* < 0.001). For men, the likelihood of having ever tested increased with age. Past use and awareness of HIVST was very low, 1.2 and 12.6%, respectively. Awareness was lower among women than men (9.1% vs 15.3%, adjusted odds ratio [aOR] = 1.55; 95% confidence interval [CI]: 1.37–1.75), and at younger ages, and lower education and literacy levels. Willingness to self-test among Zimbabwean men was high (84.5%), with greater willingness associated with having previously tested for HIV, being at high sexual risk (highest willingness [aOR = 3.74; 95%CI: 1.39–10.03, *p* < 0.009]), and being ≥25 years old. Wealthier men had greater awareness of HIVST than poorer men (*p* < 0.001). The highest willingness to self-test (aOR = 3.74; 95%CI: 1.39–10.03, *p* < 0.009) was among men at high HIV-related sexual risk.

**Conclusions:**

In 2015–16, many Malawian and Zimbabwean men had never tested for HIV. Despite low awareness and minimal HIVST experience, willingness to self-test was high among Zimbabwean men, especially older men with moderate-to-high HIV-related sexual risk. These data provide a valuable baseline against which to investigate population-level uptake of HIVST as programmes scale up. Programmes introducing, or planning to introduce, HIVST should consider including relevant questions in population-based surveys.

## Background

Both Malawi and Zimbabwe have made tremendous progress toward the “first 90” global target of diagnosing 90% of people with HIV. In 2016, estimates showed that 84% of people with HIV in Malawi and 86% in Zimbabwe were aware of their status [1]. By end-2018, 90% of all people with HIV had been diagnosed: 940000 and 1.3 million people in Malawi and Zimbabwe, respectively [1]. As a result, reaching the remaining people with HIV who do not know their status is becoming costly and challenging, with national programmes reporting declining numbers of people with HIV diagnosed through HIV testing services [2, 3]. Global and national priorities now include defining sustainable approaches that maintain these high rates of testing coverage, while reaching individuals and groups still in need of HIV testing, prevention and treatment.

Across southern Africa, men are less well served by HIV programmes than women, less likely to have ever tested [4] and more likely to develop advanced HIV disease, reflecting late diagnosis and/or treatment initiation [5]. Men have fewer opportunities for HIV testing compared to women, as well as social–cultural, economic and systemic barriers that reduce access to and uptake of services [6, 7].

HIV self-testing (HIVST) is recommended by the World Health Organization (WHO) [2] and is a key intervention for reaching populations who may not test otherwise, particularly men [8]. Results from multiple evaluations show that HIVST has a high uptake, can increase the population coverage of HIV testing, and has high safety and acceptability globally [9, 10]. As of July 2019, this recommendation has been taken up globally, with nearly 7 million HIVST kits procured by major donors, and 77 countries reporting that they have an HIVST policy, 38 of which are fully implementing self-testing [11, 12].

Both Malawi and Zimbabwe were early adopters of self-testing, with pilot studies starting between 2010 and 2015 [13, 14]. These pilots were then followed by the development of national policies and initiation of large-scale implementation in mid-2015 under the STAR (Self-Test AfRica) Initiative [15]. Since then, multiple evaluations of HIVST in each country have shown community- and facility-based HIVST, as well as partner-delivered HIVST, to be feasible and effective ways of reaching first-time testers, men, young people, as well as partners of people with HIV [10, 16–19]. Recent mathematical modelling suggests that HIVST can also be cost-effective with appropriate targeting of men in southern Africa among other priority groups [20, 21].

As both countries move toward broader scale up of self-testing, we used Demographic and Health Survey (DHS) data from 2015 to 16 to analyse population-level awareness and use of, and willingness to self-test prior to large-scale implementation [22, 23]. These questions were initially optional additions to the DHS questionnaire in 2015. As such, the objective of this study was to provide a point of comparison with future evaluations post national scale up, as well as to inform future implementation of HIVST. We assessed early implementation of HIVST questions in population-based surveys, and associations with awareness and use of, and future willingness to, self-test.

## Methods

We obtained population-based survey data from the 2015–16 Malawi and Zimbabwe DHS with standard permissions from DHS and ICF International [22, 23]. These provide data from a representative sample of men (15–54 years) and women of reproductive age (15–49 years) living in Malawi and Zimbabwe, with linked laboratory HIV test results. We limited our analysis to participants who had completed interviewer-administered questionnaires, provided blood specimens for HIV testing, and had a valid result from this HIV test.

Our main outcomes of interest were self-reported by survey respondents: ever testing for HIV, awareness and use of HIVST, and willingness to self-test in the future. Willingness to self-test was asked only in Zimbabwe, and included only in the male questionnaire. The complete survey questionnaires are accessible on the DHS website: https://dhsprogram.com/.

### Independent variables

**Fig. 1:**
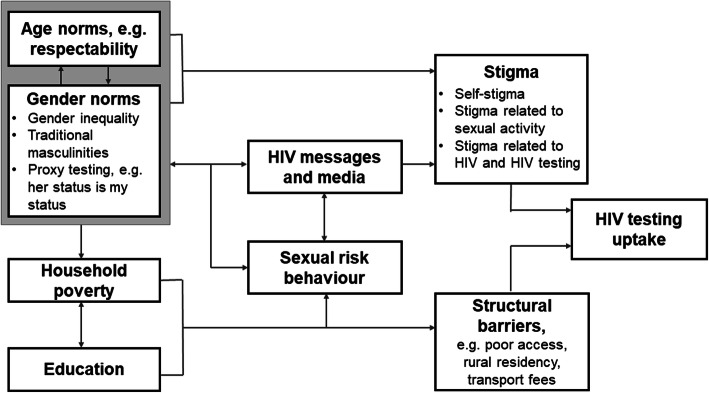
Mechanisms affecting HIV testing uptake in adults (aged 15+ years) in southern Africa, by age, gender, and sexual risk behaviour

The choice of independent covariates was informed by the literature on factors influencing testing for HIV and adaptation of the simplified hierarchical framework for HIV testing (including self-testing) among men in sub-Saharan Africa (*see* Fig. 1). We also pre-specified a stratified analysis by HIV testing history to explore differences in awareness and use of, and willingness to, self-test for HIV.

Independent variables used in the analysis included country (i.e. Malawi or Zimbabwe), sex (i.e. male or female), household wealth (i.e. measured by standard quintiles), age (i.e. measured by five-year age bands from 15 to 45 years and 45+ years), education (i.e. measured by secondary education or lower), literacy (i.e. ability to read, or not read, a full sentence), employment (i.e. actively working in the past 7 days), marital status (i.e. married or cohabiting), and HIV status reported during the survey (i.e. HIV-positive or HIV-negative). A three-category HIV-related sexual risk variable was defined from reported sexual activity (i.e. measured by sexual activity, or inactivity, in the past 4 weeks), and the following high-risk exposures in the previous 12 months: multiple (i.e. ≥2) partners; any paid sex (asked to men); having received gifts, cash or other compensation in exchange for sex (asked to women); and having a sexually transmitted infection (STI). Individuals with one or more of these risk variables were classified as “high-risk”. The remaining respondents reporting no other risk exposures were classified as “moderate risk” if sexually active in the past 4 weeks and “low-risk” if reporting no sexually activity in the past 4 weeks.

### Data analysis

We used Stata version 11 for analysis (College Station, Texas). We set standard country-specific sampling and cluster weights provided by DHS using the survey (svy) commands. We excluded participants with missing data for outcomes or independent variables from the analysis.

As independent variables were all categorical, we reported baseline characteristics as proportions. We selected variables for inclusion in multivariable models by the putative causal framework (Fig. 1), and by investigating effect modification and collinearity. Univariable and multivariable analyses used logistic regression. We calculated *p*-values across age, wealth and HIV-related sexual risk using the Wald test.

We investigated associations between independent and outcome variables using univariable odds ratios (ORs) with 95% confidence intervals (CI). Before multivariable analysis, we explored confounding and collinearity between independent variables by investigating associations between variables for all those with significant associations with any given outcomes. We explored potential effect modification [24] using stratified analyses by sex, HIV status, previous HIV testing history and HIV-related sexual risk category.

## Results

### Baseline characteristics

We included 31,385 survey respondents reporting on HIV testing history: 14,911 and 16,474 records from Malawi and Zimbabwe, and 14,027 and 17,358 among men and women, respectively (Table 1). Of these, a total of 24,683 individuals were asked about HIVST, and 6702 (21.4%, *n* = 31,385) not asked. An additional 15 individuals (0.06%, *n* = 24,683) asked about HIVST had missing data related to questions on sexual activity used to determine HIV-related sexual risk.

**Table 1:** Baseline characteristics in Malawi and Zimbabwe, 2015–16

Variables^a^	Ever tested (***N*** = 31,385)^b^			Ever self-test (***N*** = 24,683)^b^			Aware self-test (***N*** = 24,683)^b^		
	*N*	%	*p*-value^§^	*N*	%	*p*-value^§^	*n*	%	*p*-value^§^
**Total population**	24,148	76.9		287	1.2		3118	12.6	
**Country**			< 0.001			< 0.001			< 0.001
Malawi	11,726	75.4		141	1.0		1671	11.4	
Zimbabwe	12,422	78.6		146	1.5		1447	14.5	
**Sex**			< 0.001			0.008			< 0.001
Female	14,500	83.5		103	1.0		983	9.1	
Male	9648	68.8		184	1.3		2135	15.3	
**Residence**			< 0.001			< 0.001			< 0.001
Urban	7951	79.9		151	2.1		1516	21.2	
Rural	16,197	75.9		136	0.8		1602	9.1	
**Age group (years)**			< 0.001			< 0.001			< 0.001
15–19	3252	44.8		30	0.5		437	7.0	
20–24	4703	80.7		42	0.9		562	12.1	
25–29	4337	90.3		59	1.6		580	16.0	
30–34	4070	91.1		55	1.7		497	15.2	
35–39	3247	89.2		42	1.5		426	15.7	
40–44	2446	87.4		35	1.7		303	15.0	
45+	2093	80.8		24	1.1		313	14.7	
**Wealth**			< 0.001			< 0.001			< 0.001
Poorest	3697	76.4		18	0.5		246	6.4	
Poorer	4029	75.8		38	0.9		336	7.8	
Middle	4252	75.3		26	0.6		279	8.3	
Rich	5594	77.6		56	0.9		669	12.2	
Richest	6576	78.5		149	2.3		1488	22.8	
**HIV status**			< 0.001			0.108			0.029
HIV negative	20,646	75.0		249	1.1		2760	12.5	
HIV positive	2570	90.6		38	1.5		358	10.4	
**Marital status**			< 0.001			0.005			< 0.001
Single	7595	58.9		99	0.9		1175	11.2	
Married or cohabiting	16,553	89.5		188	1.3		1943	13.7	
**Employment**			< 0.001			0.033			< 0.001
Not actively working	8719	70.1		85	1.0		823	9.4	
Actively working	15,429	81.4		202	1.3		2295	14.4	
**Education**			< 0.001			< 0.001			< 0.001
≤ Primary	10,617	74.8		70	0.6		927	7.5	
≥ Secondary	13,531	78.7		217	1.8		2191	17.8	
**Literacy**			< 0.001			< 0.001			< 0.001
Illiterate	5211	73.5		44	0.7		488	8.0	
Literate	18,937	77.9		243	1.3		2630	14.2	
**Sexually active**			< 0.001			< 0.001			< 0.001
Sexually inactive	9064	64.0		107	1.5		1225	17.7	
Active in past 4 weeks	15,053	87.6		180	1.5		1890	14.2	
**HIV-related risk**^c^			< 0.001			< 0.001			< 0.001
Low risk	8457	63.5		94	0.9		1102	10.3	
Moderate risk	13,092	88.7		134	1.2		1497	13.6	
High risk	2570	78.8		59	2.0		516	17.2	

^a^Ever tested refers to people surveyed on HIV testing history who reported that they previously tested for HIV before the survey. Overall, 31,385 people were asked about their HIV testing history and 24,148 responded that they had tested previously. Ever self-tested refers to people surveyed on HIV self-testing who reported that they had previously self-tested. Overall, 24,683 people were asked whether they had self-tested and 287 reported that they had self-tested previously. Aware of self-testing refers to people surveyed who reported that they were aware of HIV self-testing. Overall, 24,683 people were asked whether they were aware of self-testing and 3118 reported that they were aware of self-testing

^b^Out of 31,385 people surveyed, 31,348 were included as 37 people were missing information on sexual activity and HIV-related risk. Not all participants were systematically surveyed on self-testing questions. Out of 31,385 people surveyed, 24,683 were asked about self-testing, resulting in a smaller sample size. Among these were 15 people reporting on self-testing who did not provide information on sexual activity and HIV risk. Population size asked about ever testing for HIV: 31347 (HIV risk/sexual activity). Population size asked about awareness or ever self-testing for HIV: 24668 (HIV risk/sexual activity)

^c^HIV risk as defined in this analysis includes reported sexual activity in the past four weeks, and the following high-risk exposures in the previous 12 months: multiple (i.e. ≥2) partners, any paid sex (asked to men), having received gifts, cash or other compensation in exchange for sex (asked to women), and having a sexually transmitted infection (STI). Individuals with any “high-risk” exposures were classified as “high-risk”, with the remaining respondents classified as “low risk” if reporting no sexually activity in the past four weeks, and as “moderate risk” otherwise

^§^*P*-value based on cluster-adjusted chi-squared test

A total of 78.6% and 75.4% of people reported ever having tested for HIV in Zimbabwe and Malawi, respectively (*p* < 0.001). More women compared to men (83.5% vs 68.8%; *p* < 0.001), and more urban compared to rural residents (79.9% vs 75.9%; *p* < 0.001) had tested previously. A larger proportion of those never tested were 15–24 years compared to those who were ≥ 25 years (see Table 1).

The proportion of people who had ever self-tested was 1.2% and similar in both countries. However, while overall 12.6% had awareness of HIVST, it was greater in Zimbabwe compared to Malawi (14.5% vs 11.4%; *p* < 0.001) and among men compared to women (15.3% vs 9.1%; *p* < 0.001) (Table 1). Among the respondents, those with greater awareness of self-testing were ≥ 30 years of age (≥30 years: 21.1% vs < 30 years: 9.1%; *p* < 0.001), wealthier (richest: 22.8% vs poorest: 6.4%; *p* < 0.001) and those with higher education levels (at least secondary education: 17.8% vs primary education or less: 7.5%; *p* < 0.001) than those aged < 30 years, those who were poorer and had lower education levels.

Willingness to self-test could be assessed only among 7372 Zimbabwean men (48 men had missing data on willingness), as only men were asked about willingness to self-test, and this question was not included in the Malawi DHS questionnaire.

Most Zimbabwean men (84.5%) were willing to self-test (Supplementary Table S1 includes baseline characteristics of Zimbabwean men on willingness to self-test, 2015–16). Men aged ≥25 years reported greater willingness to self-test than men aged < 25 years (88.7% vs 78.8%; *p* < 0.001). High-risk men also reported greater willingness to self-test than low-risk men (78.8% vs 63.5%; *p* < 0.001). Most men willing to self-test had tested in the past 12 months (88.5%). However, 86.4% of the men who had not tested for HIV in the previous two or more years were also willing to self-test.

### Ever testing for HIV

Age, HIV status and HIV-related sexual risk appeared to modify effects in the multivariable analysis across a number of variables **(**Table 2**)**. Collinearity affected the results of multivariable analysis, notably between age and HIV-related sexual risk, marital status and HIV-related sexual risk, age and education level, and education level and literacy.

**Table 2:** Univariable and multivariable associations between sociodemographic factors and ever testing for HIV in Malawi and Zimbabwe, 2015–16

Variables	Univariable (weighted) ***N*** = 31,375^a^		Multivariable (weighted) ***N*** = 31,347^a^	
	OR	95% CI and *p*-value	aOR	95% CI and *p*-value
**Country**				
Zimbabwe	1		1	
Malawi	1.18	1.10–1.28	1.26	1.15–1.38
**Sex**				
Female	1		1	
Male	0.42	0.3–0.45	0.39	0.36–0.42
**Age (years)**				
15–19	1	*p* < 0.001^§^	1	*p* < 0.001^§^
20–24	5.40	4.87–5.98	4.37	3.91–4.87
25–29	11.89	10.48–13.48	8.24	7.18–9.46
30–34	14.19	12.38–16.27	8.86	7.63–10.29
35–39	10.42	9.01–12.05	6.41	5.47–7.50
40–44	8.44	7.27–9.80	5.30	4.50–6.25
45+	5.16	4.50–5.91	3.72	3.20–4.34
**Residence**				
Urban	1		1	
Rural	0.83	0.76–0.90	1.00	0.88–1.14
**HIV status**				
HIV negative	1		1	
HIV positive	3.44	2.98–3.97	2.11	1.80–2.49
**Marital status**				
Single	1		1	
Married or cohabiting	6.07	5.62–6.54	^b^	^b^
**Wealth**				
Poorest	1	*p* < 0.003^§^	1	*p* < 0.581^§^
Poor	0.94	0.84–1.05	1.02	0.89–1.16
Middle	0.91	0.81–1.01	1.09	0.96–1.25
Rich	1.04	0.93–1.16	1.05	0.91–1.20
Richest	1.09	0.97–1.23	1.11	0.94–1.31
**Employment**				
Not actively working	1		1	
Actively working	1.81	1.69–1.95	1.16	1.06–1.26
**Education**				
≤ Primary	1		1	
≥ Secondary	1.26	1.17–1.35	^b^	^b^
**Literacy**				
Illiterate	1		1	
Literate	1.30	1.20–1.40	1.63	1.50–1.78
**HIV-related risk**^c^				
Low risk	1	*p* < 0.001^§^	1	*p* < 0.001^§^
Moderate risk	4.58	4.23–4.96	2.15	1.96–2.36
High risk	2.14	1.92–2.40	1.54	1.80–2.49

^a^ Both samples were weighted based on standard DHS weights; Strata = 56; PSU = 1256. Univariable draws from a total of 31,385 observations, population size 31,375. Multivariable draws from a total of 31,348 observations and population size of 31,338. This excludes 37 people who did not report on sexual activity and risk behaviours and are missing from the “HIV risk category”

^b^Represents variables that were not included in the multivariable analysis due to identified collinearity

^c^ HIV risk as defined in this analysis includes reported sexual activity in the past four weeks, and the following high-risk exposures in the previous 12 months: multiple (i.e. ≥2) partners, any paid sex (asked to men), having received gifts, cash or other compensation in exchange for sex (asked to women), and having a sexually transmitted infection (STI). Individuals with any “high-risk” exposures were classified as “high-risk”, with the remaining respondents classified as “low risk” if reporting no sexually activity in the past four weeks, and as “moderate risk” otherwise

^§^*P*-value based on the Wald test. *P*-values for variables with more than two categories are shown

On multivariable analysis, after assessing for collinearity, being Malawian was associated with ever having tested for HIV (adjusted odds ratio [aOR] = 1.26; 95% confidence interval [CI]: 1.15–1.38, *p* < 0.001) (Table 2). However, men had substantially lower odds of having ever tested for HIV compared to women (aOR = 0.39; 95%CI: 0.36–0.42, *p* < 0.001). Individuals between 30 and 34 years of age had greater odds of ever having tested for HIV compared to 15–19 year olds (aOR = 8.86; 95%CI: 7.63–10.29, *p* < 0.001). Additional factors associated with ever having tested for HIV included: an HIV-positive test result in the survey (HIV-positive vs HIV-negative: aOR = 2.11, 95%CI: 1.80–2.49, *p* < 0.001), employment (actively working vs not actively working: aOR = 1.16; 95%CI: 1.06–1.26, *p* < 0.001), literacy (being literate vs being illiterate: aOR = 1.63, 95%CI: 1.50–1.78, *p* < 0.001), and reporting more HIV-related sexual risk behaviours (moderate vs low: aOR = 2.15; 95%CI: 1.96–2.36, *p* < 0.001, and high-risk vs low-risk: aOR = 1.54; CI: 1.80–2.49, *p* < 0.001).

### Use and awareness of self-testing

A complete analysis of ever self-testing is shown in supplementary Table S2. Table 3 provides outcomes from the univariable and multivariable analyses for awareness of HIV self-testing.

**Table 3:** Univariable and multivariable associations between sociodemographic factors and awareness of HIV self-testing in Malawi and Zimbabwe, 2015–16

Variables	Univariable (weighted) ***N*** = 24,683^a^		Multivariable (weighted) ***N*** = 24,668^a^	
	OR	95% CI and *p*-value	aOR	95% CI and *p*-value
**Country**				
Zimbabwe	1		1	
Malawi	0.76	0.67–0.87	0.82	0.70–0.94
**Sex**				
Female	1		1	
Male	1.73	1.54–1.92	1.55	1.37–1.75
**Age**				
15–19	1	*p* < 0.001^§^	1	*p* < 0.001^§^
20–24	1.79	1.50–2.12	1.35	1.12–1.62
25–29	2.52	2.11–3.00	1.76	1.43–2.17
30–34	2.44	2.02–2.94	1.66	1.32–2.08
35–39	2.46	2.04–2.97	1.69	1.34–2.12
40–44	2.09	1.70–2.55	1.45	1.14–1.86
45+	2.00	1.64–2.46	1.31	1.04–1.66
**Residence**				
Urban	1		1	
Rural	0.33	0.29–0.39	0.64	0.55–0.77
**Ever tested**				
No	1		1	
Yes	2.18	1.94–2.45	1.89	1.65–2.17
**HIV status**				
HIV negative	1		1	
HIV positive	1.12	0.95–1.31	0.89	0.75–1.06
**Marital status**				
Single	1		1	
Married or cohabiting	1.26	1.13–1.39	^b^	^b^
**Wealth**				
Poorest	1	*p* < 0.001^§^	1	*p* < 0.001^§^
Poor	1.26	1.04–1.53	1.24	1.02–1.51
Middle	1.26	1.03–1.53	1.25	1.02–1.53
Rich	1.87	1.53–2.28	1.49	1.20–1.84
Richest	4.30	3.54–5.22	3.03	2.46–3.73
**Employment**				
Not actively working	1		1	
Actively working	1.63	1.47–1.82	1.25	1.12–1.42
**Education**				
≤ Primary	1		1	
≥ Secondary education	2.69	2.38–3.04	^b^	^b^
**Literacy**				
Illiterate	1		1	
Literate	1.84	1.59–2.12	1.17	1.01–1.36
**HIV risk**^c^				
Low risk	1	*p* < 0.001^§^	1	*p* < 0.518
Moderate risk	1.37	1.24–1.53	1.03	0.90–1.17
High risk	1.75	1.51–2.03	1.10	0.93–1.31

^a^ Both samples were weighted based on standard Demographic and Health Survey weights; Strata = 56; PSU = 1256. Not all participants were systematically surveyed on self-testing questions. Out of 31,385 people surveyed, 24,683 were asked about self-testing, resulting in a smaller sample size. Among those reporting on HIV self-testing, 15 did not provide information on sexual activity and HIV risk. Population size asked about awareness or ever self-testing for HIV: 24668 (HIV risk), 24,668 (sexual activity)

^b^ Represents variables that were not included in the multivariable analysis due to identified collinearity

^c^ HIV risk as defined in this analysis includes reported sexual activity in the past four weeks, and the following high-risk exposures in the previous 12 months: multiple (i.e. ≥2) partners, any paid sex (asked to men), having received gifts, cash or other compensation in exchange for sex (asked to women), and having a sexually transmitted infection (STI). Individuals with any “high-risk” exposures were classified as “high-risk”, with the remaining respondents classified as “low risk” if reporting no sexually activity in the past four weeks, and as “moderate risk” otherwise

^§^*P*-value based on the Wald test. *P*-values for variables with more than two categories are shown

In the multivariable analysis, men aged 30–34 years had greater odds of past self-testing use compared to younger men (age 15–19 years) (aOR = 2.89; 95%CI: 1.47–5.68, *p* < 0.002) (**Table****S2**). Across wealth quintiles, being wealthier was also associated with previous self-testing (*p* < 0.001), with the wealthiest individuals having the greatest odds of past self-testing (aOR for richest vs poorest = 3.59; 95%CI: 1.79–7.18, *p* < 0.001).

In the multivariable analysis, respondents in Malawi and those from a rural setting were less likely to be aware of HIVST compared with Zimbabweans and urban participants (Table 3). However, the following variables were significantly associated with being aware of HIVST: being male (male vs female: aOR = 1.55; 95%CI: 1.37–1.75, *p* < 0.001), aged 15–19 years (when compared with those aged 25–29 years: aOR = 1.76; 95%CI: 1.43–2.17, *p* < 0.001 and aged 35–39 years: aOR = 1.69; 95%CI: 1.34–2.12, *p* < 0.001), wealthier (wealthiest vs poorest: aOR = 3.03; 95%CI: 2.46–3.73, *p* < 0.001), having employment (actively working vs not actively working: aOR = 1.25; 95%CI: 1.12–1.42, *p* < 0.001), being literate (literate vs illiterate: aOR = 1.17; 95%CI: 1.01–1.36, *p* < 0.035) and having previously tested for HIV (ever tested vs never tested: aOR = 1.89; 95%CI: 1.65–2.17, *p* < 0.001).

### Willingness to self-test among Zimbabwean men

The relationship between willingness to test and socioeconomic variables (wealth and actively working) and HIV status substantially differed according to both high and low HIV-related sexual risk (Table 4): *se*e, for example, univariable OR for HIV status and employment. Thus, we adapted our planned multivariable analysis to account for effect modification between HIV-related sexual risk categorization and socioeconomic variables. On multivariable analysis, men with high HIV-related sexual risk behaviours were more likely than low-risk men to express willingness to self-test if they were also from higher socioeconomic quintiles, not working, in rural settings and had tested previously (interaction terms: socioeconomic status, *p* = 0.066; rural residence, *p* = 0.071; employment *p* = 0.003; literacy, *p* = 0.225; married, *p* = 0.401; aware of self-test, *p* = 0.605; previous testing, *p* = 0.001; and HIV status *p* = 0.162).

**Table 4:** Univariable and multivariable associations between sociodemographic factors and willingness to self-test among men in Zimbabwe, by those at low, moderate and high HIV-related risk, 2015–16

Variables	Univariable (weighted)						Multivariable (weighted)					
	Having low risk (***n*** = 3142)^a^		Having moderate risk (***n*** = 2988)^a^		Having high risk (***n*** = 1241)^a^		Having low risk (***n*** = 3142)^a^		Having moderate risk (***n*** = 2988)^a^		Having high risk (***n*** = 1241)^a^	
	OR	95% CI and *p*-value	OR	95% CI and *p*-value	OR	95% CI and *p*-value	aOR	95% CI and *p*-value	aOR	95% CI and *p*-value	aOR	95% CI and *p*-value
**Age (years)**												
15–19	1	*p* < 0.001^§^	1	*p* = 0.063^§^	1	*p* = 0.028^§^	1	*p* = 0.106^§^	1	*p* = 0.343^§^	1	*p* = 0.030^§^
20–24	1.78	1.37–2.30	1.49	0.70–3.23	2.44	1.21–4.92	1.47	1.11–1.92	1.31	0.60–2.85	2.71	1.32–5.57
25–29	2.00	1.36–2.96	2.43	1.18–4.99	2.09	0.99–4.41	1.50	1.00–2.27	1.89	0.90–3.95	2.66	1.23–5.75
30–34	2.01	1.11–3.63	2.52	1.24–5.09	2.86	1.35–6.05	1.44	0.79–2.64	1.98	0.96–4.07	3.82	1.82–8.00
35–39	1.69	0.96–2.99	2.31	1.10–4.85	3.77	1.75–9.14	1.17	0.65–2.10	1.92	0.91–4.07	4.87	2.14–11.07
40–44	1.82	0.88–3.78	2.52	1.16–5.44	2.21	0.95–5.16	1.27	0.59–2.72	2.09	0.96–4.59	3.02	1.18–7.71
45+	1.61	0.93–2.81	1.70	0.87–3.33	1.94	0.93–4.07	1.05	0.56–1.95	1.44	0.72–2.88	2.46	1.09–5.54
**Residence**												
Urban	1		1		1		1		1		1	
Rural	0.81	0.64–1.02	1.18	0.89–1.55	1.33	0.86–2.06	0.71	0.49–1.03	1.14	0.74–1.76	3.56	1.61–7.90
**Wealth**												
Poorest	1	*p* = 0.128^§^	1	*p* = 0.113^§^	1	*p* = 0.981^§^	1	*p* = 0.102^§^	1	*p* = 0.260^§^	1	*p* = 0.080^§^
Poor	1.04	0.75–1.45	1.87	1.13–3.09	1.16	0.58–2.30	1.02	0.74–1.41	1.72	1.02–2.91	1.27	0.64–2.50
Middle	0.97	1.00–1.90	1.27	0.85–1.88	0.96	0.49–1.89	0.98	0.71–1.35	1.20	0.78–1.84	1.04	0.51–2.10
Rich	1.38	0.70–1.34	1.12	0.71–1.77	1.03	0.52–2.05	1.04	0.73–1.47	1.03	0.60–1.77	2.64	1.07–6.53
Richest	1.02	0.74–1.42	1.12	0.75–1.67	1.10	0.57–2.12	0.65	0.42–1.02	1.02	0.54–1.94	3.74	1.39–10.03
**Employment**												
Not actively working	1		1		1		1		1		1	
Actively working	1.64	1.35–1.99	1.19	0.86–1.63	0.72	0.44–1.18	1.41	1.13–1.77	1.12	0.78–1.61	0.57	0.34–0.95
**HIV status**												
HIV negative	1		1		1		1		1		1	
HIV positive	1.82	1.19–2.79	0.94	0.56–1.59	0.76	0.43–1.35	1.41	0.87–2.30	0.84	0.49–1.42	0.67	0.37–1.21
**Marital status**												
Single	1		1		1		1		1		1	
Married or cohabiting	0.59	0.40–89	0.72	0.47–1.10	0.72	0.49–1.06	^b^	^b^	^b^	^b^	^b^	^b^
**Education**												
≤ Primary	1		1		1		1		1		1	
≥ Secondary	1.52	1.22–1.89	1.20	0.91–1.58	1.19	0.77–1.86	^b^	^b^	^b^	^b^	^b^	^b^
**Literacy**												
Illiterate	1		1		1		1		1		1	
Literate	1.23	0.98–1.55	1.66	1.18–2.32	1.36	0.83–2.22	1.16	0.91–1.48	1.55	1.07–2.25	1.32	0.78–2.23
**Ever tested**												
No	1		1		1		1		1		1	
Yes	1.74	1.40–2.15	2.00	1.47–2.72	1.40	0.88–2.20	1.48	1.18–1.85	1.87	1.37–2.55	1.20	0.76–1.90
**Aware of self-test**												
No	1		1		1		1		1		1	
Yes	1.35	0.95–1.92	0.96	0.69–1.34	1.00	0.56–1.78	1.09	0.76–1.55	0.94	0.66–1.33	0.89	0.50–1.60

^a^ Weighted analysis using standard Demographic and Health Survey (DHS) sample weights: Sample size = 7041.0867; Strata = 19; PSU = 400. Out of 7420 men surveyed, 7372 reported on willingness to self-test. Forty-eight men did not respond and one did not provide information on sexual activity (HIV risk). Sexual activity was not reported by one respondent and could not be used in the HIV risk variable. These variables have a total sample size of 7371

^b^ Represents variables that were not included in the multivariable analysis due to identified collinearity

^c^ HIV risk as defined in this analysis includes reported sexual activity in the past four weeks, and the following high-risk exposures in the previous 12 months: multiple (i.e. ≥2) partners, any paid sex (asked to men), having received gifts, cash or other compensation in exchange for sex (asked to women), and having a sexually transmitted infection (STI). Individuals with any “high-risk” exposure were classified as “high-risk”, with the remaining respondents classified as “low risk” if reporting no sexually activity in the past four weeks, and as “moderate risk” otherwise

^§^*P*-value based on the Wald test. *P*-values for variables with more than two categories are shown

On multivariable analysis of men at high HIV-related sexual risk, willingness to self-test increased with age (*p* = 0.030), with the strongest association for those aged 35–39 years compared to those aged 15–19 years (aOR = 4.87; 95%CI: 2.14–11.07, *p* < 0.001). Similarly, willingness to self-test among men with high HIV-related sexual risk increased in rural settings (rural vs urban: aOR = 3.56, 95%CI: 1.61–7.90, *p* = 0.002) and with greater wealth quintiles (wealthiest vs least wealthy: aOR = 3.74, 95%CI: 1.39–10.53, *p* = 0.009).

While actively working men with high HIV-related risk were less willing to self-test (actively working vs not actively working: aOR: 0.57, 95%CI: 0.34–0.95, *p* = 0.030), actively working low-risk men were more willing to self-test than when not actively working (aOR 1.41; 95%CI: 1.13–1.77, *p* = 0.003). The association with previous testing and willingness to test was also more pronounced for low-risk men (ever tested vs never tested: aOR 1.48; 95%CI: 1.18–1.85, *p* < 0.001) than high-risk men (ever tested vs never tested: aOR 1.20; 95%CI: 0.76–1.90, *p* = 0.435), while associations with age (*p* = 0.106) and wealth (*p* = 0.102) were less pronounced than for high-risk men (Table 4, described above).

We additionally conducted a stratified analysis to investigate whether willingness to self-test varied by past HIV-testing behaviour (i.e. previously tested or not) (*see* supplementary Table S3). Patterns of willingness to self-test were similar for the 2437/7372 (33.1%) men who had never previously tested as for those with at least one past HIV test, with greater willingness in older men.

## Discussion

The main findings from this analysis of 2015–16 survey data captured immediately before HIVST implementation in Malawi and Zimbabwe were that awareness and lifetime use of self-testing were low, with 12.6% of respondents being aware of self-testing and 1.2% having ever self-tested for HIV. Willingness to self-test was high, although this question was asked only of male Zimbabweans, with 84.5% respondents reporting themselves willing, including 30.4% of all previously untested men. Self-testing appeared to appeal most strongly to older men and those with high-to-moderate HIV-related sexual risk. The highest willingness to self-test was in men aged 35–39 years and those in rural settings, where having never previously tested for HIV was more common than in urban settings. Factors independently associated with greater awareness of HIVST included men, urban residence, and literacy; with many of these same factors also associated with having tested for HIV at least once in this analysis of 2015–16 data. Poorer and unemployed individuals were less likely to be aware of self-testing.

Despite significant gains and scale up of HIV testing in both Malawi and Zimbabwe, men continue to be missed [1, 2]. According to recent “first 90” estimates in sub-Saharan Africa, the absolute number of men with HIV aged ≥25 years are much less likely to know their HIV-positive status than women overall and younger men [25]. As the median age of all people with HIV continues to increase [26], identifying and scaling-up strategies that appeal to older age groups will be needed, especially older men and those at high risk. Greater efforts are needed to roll out evidence-based HIVST approaches to reaching men, such as through health facilities and secondary distribution from female partners attending antenatal care in high HIV-burden settings, or through networks of other high-risk sexual, drug injecting or social contacts, including those with HIV [9, 16, 27, 28].

Considering the high willingness to self-test in high-risk men in rural areas, additional community outreach strategies may be needed. HIVST in workplaces and through faith-based organizations should also be considered, as early programmatic data suggest it may be particularly useful for reaching older men [29]. However, more focused programmatic efforts and communication strategies for workplace HIVST may be needed, as in contrast to low-risk men, high-risk men who were working were less willing to self-test. Further evaluation is needed to understand the utility of HIVST through formal and informal workplace programmes and how well they can reach high-risk men. It will be important to assess differences in HIVST awareness, use and willingness among older and higher-risk men in future surveys.

The importance of high willingness to self-test among older Zimbabwean men, including those with higher risk factors, should not be underestimated. This challenges perceptions that men may not want to test or are afraid to test for HIV and underscores the importance of providing more opportunities and HIV testing options that are acceptable to men. As reported in a recent analysis among never tested men in sub-Saharan Africa, nearly all those offered HIV testing in the survey accepted it and learnt their results [4].

Since these surveys, HIVST, alongside conventional testing, has been rapidly scaled up, notably so for Malawi and Zimbabwe. Between May 2015 and July 2017, the STAR Initiative alone distributed 172,830 and 265,091 HIVST kits in Malawi and Zimbabwe, respectively [10]. Following publication of the WHO guidelines and WHO prequalification of four HIVST products, as well as multiple large-scale implementation studies [2, 30], volumes continue to increase annually, with latest estimates suggesting that between 2017 and 2020, with existing donor support, both countries will have procured at least 4 million self-testing kits [12].

High willingness to self-test in Malawi and Zimbabwe has also been underscored by the observed high uptake in community-based HIVST interventions. Uptake by 45–75% was reported by end-line surveys between 2016 and 2019 in three population-level cluster randomized trials in rural communities [18, 19, 31]. In 2017, a survey following community-based HIVST kit distribution in rural Zimbabwe, with or without supply-side financial incentives for post-test linkage, showed that 81.7% of residents were aware of self-testing and 55.8% had self-tested [18]. Two trials of community distribution of HIVST kits in rural Malawi showed high uptake of HIVST, with significant increase in ever testing for HIV in men and adolescents [19, 31]. Even in the standard-of-care arms, 31.5% of participants in the 2016–17 trial and 32.3% in the 2018–19 trial, respectively, were aware of HIVST [18, 19, 31].

These are substantial increases compared to the low awareness and use of self-testing in the 2015–16 DHS, and highlight the broader impact on awareness from large implementation science studies, such as the STAR Initiative. In 2015–16, HIVST was limited to small pilot studies in each country, as national and international policies were still under development and there were no nationally registered or WHO-prequalified products available [32].

As HIVST continues to expand globally, monitoring overall HIVST use, and awareness of and willingness to test will contribute to a better understanding of the reach and impact of HIVST. Ideally, the extent to which social determinants such as urban residence, literacy and affluence dictate awareness of HIVST will diminish with more comprehensive distribution strategies such as those through community outreach, health facilities, by sexual partners and in other venues such as workplaces and private sector pharmacies. Population-based surveys, like the DHS, will then provide an important source of information for countries implementing HIVST, as well as those planning to add HIVST as part of existing HIV testing services. Together with routine programmatic data and special studies, population-based surveys that have included questions on HIVST can then provide a meaningful baseline and point of comparison for future analyses and important insights for future implementation.

Although Malawi and Zimbabwe have scaled up HIV testing and have now achieved the first “90”, gaps remain, particularly among men. Efforts are on to reach the first “95” by 2030 – diagnosing 95% of all people with HIV – which is the new goal. As a result, strategies for diagnosing the shrinking number of people with HIV who do not know their status are becoming more challenging and also less cost-effective unless targeted toward specific populations and settings with lower knowledge of status among people with HIV [3]. Maintaining the high testing coverage and knowledge of status achieved will not be inexpensive and HIVST is likely to play a role in sustaining services and potentially reducing costs. Furthermore, HIVST also addresses patient costs of accessing services and equity concerns, which also need to be considered, especially as programmes get closer to the national goals.

Programmes will need to carefully evaluate how they can both maintain essential HIV testing services in facilities, while also deploying highly focused and effective outreach with limited resources. Strategies such as offering HIVST through specific channels among priority populations, or through periodic and geographically targeted community outreach (such as every 5 years), may be more cost-effective and affordable as more people with HIV learn their status and new infections decline [20].

## Limitations

This study has many strengths, such as its large sample size and that it is one of the first to provide an assessment of HIVST use and awareness of, and willingness to, self-test in two early-adopter African countries prior to wide-scale implementation. As such, it provides insight into the progress and changes made since HIVST has been rolled out, serving as an example for countries monitoring HIVST implementation and scale up. Pooling results, however, may have limited the ability to analyse some differences between countries.

As a cross-sectional survey using self-reported information, there may be reporting bias due to social desirability [33]. Previous studies have highlighted challenges with collecting self-reported data, particularly related to sexual risk behaviours and HIV testing history [34, 35]. Thus, it is possible that there may be differences between what people reported and their actual behaviour. Given that HIVST was relatively new during the surveys, it is possible that willingness may also change as more people have experience self-testing. Additionally, few respondents reported awareness of and past self-testing, which may introduce bias and affect the reliability of the results. It will be important to assess awareness and use of self-testing, as well as willingness to self-test in the future, following broader implementation and scale up.

Like many population-based surveys, the respondents included were limited to women 15–49 years old and men 15–54 years old. Efforts will be needed to consider older populations, particularly as the median age of people with HIV increases. Also, given that we included two of the first countries to include questions on HIVST, there were discrepancies in implementation, such that not all those surveyed were asked about self-testing, and willingness to self-test could not be assessed in Malawians or Zimbabwean women. Willingness to self-test may be similar or different among women and among Malawians, and it will be important to ensure that their replies to these questions are included in future surveys.

## Conclusions

Even in 2019, the percentage of people who had never tested for HIV remained above target for Malawian and Zimbabwean men aged ≥25 years [25]. Reaching these men will be critical to achieving the 2030 goals and maintaining low HIV incidence. Despite low awareness and previous use of HIVST among 2015–16 DHS respondents, willingness to self-test was high, especially among older Zimbabwean men with high sexual risk. Reaching these groups is a priority for HIV testing, prevention and care services as we move towards HIV elimination. Social determinants – notably urban residence, paid employment, literacy and wealth – had a pronounced impact on awareness of HIVST in 2015–16, a time that preceded programmatic implementation.

These data provide a valuable baseline against which to investigate population-level HIVST uptake and equity as programmes scale up. Countries conducting population-based surveys, especially those where HIVST is being used or is soon to be introduced, should consider including questions to assess knowledge and awareness of, and willingness to self-test, with the aim of providing baseline data, and to better understand the potential impact of HIVST over time and across and within countries.

## Supplementary information

**Additional File 1: Table S1.** Baseline characteristics of men in Zimbabwe reporting on willingness to self-test, 2015–16. Supplementary data with baseline characteristics of men in Zimbabwe reporting on willingness to self-test

**Additional File 2: Table S2.** Univariable and multivariable associations between sociodemographic factors and ever-self-testing for HIV in Malawi and Zimbabwe, 2015–16. Supplementary data with univariable and multivariable associations between sociodemographic factors and ever-self-testing for HIV in Malawi and Zimbabwe

**Additional File 3: Table S3.** Univariable and multivariable associations between sociodemographic factors and willingness to self-test among men in Zimbabwe, by testing history, 2015–16. Supplementary data with univariable and multivariable associations between sociodemographic factors and willingness to self-test among men in Zimbabwe

## Data Availability

We obtained required permissions from DHS and accessed data from the DHS website. All data and materials used in this analysis are available through the DHS programme: https://dhsprogram.com/.

## Abbreviations

aOR: Adjusted odds ratio
CI: Confidence internal
DHS: Demographic and health survey
HIV: Human immunodeficiency virus
HIVST: HIV self-testing
IRB: International review board
OR: Odds ratio
STAR: Self-testing AfRica
STI: Sexually transmitted infection
WHO: World health organization
