# Supplementary material for: Use and awareness of and willingness to self-test for HIV: an analysis of cross-sectional population-based surveys in Malawi and Zimbabwe
Source: BMC Public Health. 2020 May 25;20:779. doi: 10.1186/s12889-020-08855-7 (PMC7249304; doi:10.1186/s12889-020-08855-7)
Supplement: Supplementary file 1 — Additional File 1: Table S1. Baseline characteristics of men in Zimbabwe reporting on willingness to self-test, 2015–16. Supplementary data with baseline characteristics of men in Zimbabwe reporting on willingness to self-test [file 12889_2020_8855_MOESM1_ESM.docx]

**Table S1. Baseline characteristics of men in Zimbabwe reporting on willingness to self-test, 2015-16**

| **Variables** | **Willing to self-test (N=7372)*** | | |
| --- | --- | --- | --- |
|  | n | % willing | p-value^§^ |
| **Population** | 6 232 | 84.5 |  |
| **Age group (yrs)** |  |  | <0.001 |
| 15-19 | 1 388 | 74.6 |  |
| 20-24 | 1 043 | 85.1 |  |
| 25-29 | 882 | 87.7 |  |
| 30-34 | 881 | 90.2 |  |
| 35-39 | 715 | 89.8 |  |
| 40-44 | 615 | 90.2 |  |
| 45+ | 708 | 85.8 |  |
| **Residence** |  |  | 0.179 |
| Urban | 2 466 | 85.2 |  |
| Rural | 3 766 | 84.1 |  |
| **Wealth quintile** |  |  | 0.068 |
| Poorest | 898 | 83.1 |  |
| Poor | 1 020 | 84.9 |  |
| Middle | 1 119 | 82.8 |  |
| Richest | 1 560 | 86.2 |  |
| Richest | 1 635 | 84.7 |  |
| **Marital status** |  |  | <0.001 |
| Single | 2 877 | 79.8 |  |
| Married or cohabiting | 3 355 | 89.1 |  |
| **Employment** |  |  | <0.001 |
| Not currently working | 2 035 | 79.8 |  |
| Actively working | 4 197 | 87.0 |  |
| **Education** |  |  | <0.001 |
| ≥Primary | 1 391 | 81.0 |  |
| ≥Secondary | 4 841 | 85.6 |  |
| **Literacy** |  |  | <0.001 |
| Being illiterate | 919 | 85.5 |  |
| Being literate | 5 313 | 79.4 |  |
| **HIV status** |  |  | 0.018 |
| HIV negative | 5 460 | 84.2 |  |
| HIV positive | 772 | 87.2 |  |
| **Sexually active**** |  |  | <0.001 |
| Not sexually active | 2 727 | 79.5 |  |
| Active in last 4-weeks | 3 505 | 89.0 |  |
| **Circumcision**** |  |  | 0.019 |
| Uncircumcised | 5 247 | 85.0 |  |
| Circumcised | 990 | 82.4 |  |
| **HIV-related risk***** |  |  | <0.001 |
| Low-risk | 2 477 | 78.8 |  |
| Moderate-risk | 2 668 | 89.3 |  |
| High-risk | 1 087 | 87.6 |  |
| **Ever tested for HIV** |  |  | <0.001 |
| No | 1 895 | 77.8 |  |
| Yes | 4 337 | 87.9 |  |
| **Last HIV test (months)**** |  |  | 0.114 |
| < 12 months | 3 016 | 88.5 |  |
| ≥ 12 months | 390 | 86.5 |  |
| ≥ 24 months | 917 | 86.4 |  |
| **Aware of HIV self-testing** |  |  | 0.010 |
| No | 5 165 | 84.1 |  |
| Yes | 1 067 | 87.0 |  |
| **Ever self-tested for HIV** |  |  | 0.001 |
| No | 6 116 | 84.3 |  |
| Yes | 116 | 95.9 |  |

***** Weighted analysis using standard Demographic and Health Survey (DHS) sample weights: Sample size = 7041.0867; Strata = 19; PSU=400. Willingness to self-test refers to people surveyed who reported they were willing to self-test in the future. Out of 7420 men surveyed, 7372 reported on willingness to self-test. 48 men did not respond and 1 not provide information on sexual activity (HIV risk). Sexual activity was not reported by 1 respondent and could not be used in HIV risk variable. These variables have a total sample size of 7371.

**Sexual activity and HIV risk reported by 7371 people, as 1 person did not provide information on sexual activity and risk. Month of last test was reported by 4920 people, as not all those surveyed had tested previously.

******* HIV risk is defined in this analysis includes reported sexual activity in the past four weeks, and the following high-risk exposures in the previous 12 months: multiple (i.e. ≥2) partners, any paid sex (asked to men), having received gifts, cash or other compensation in exchange for sex (asked to women), and having a sexually transmitted infection (STI). Individuals with any “high-risk” exposures were classified as “high-risk”, with the remaining respondents classified as “low risk” if reporting no sexually activity in the past four weeks, and as “moderate risk” otherwise.

^§^ P-value based on cluster-adjusted chi-squared test.
