# Supplementary material for: Use and awareness of and willingness to self-test for HIV: an analysis of cross-sectional population-based surveys in Malawi and Zimbabwe
Source: BMC Public Health. 2020 May 25;20:779. doi: 10.1186/s12889-020-08855-7 (PMC7249304; doi:10.1186/s12889-020-08855-7)
Supplement: Supplementary file 2 — Additional File 2: Table S2. Univariable and multivariable associations between sociodemographic factors and ever-self-testing for HIV in Malawi and Zimbabwe, 2015–16. Supplementary data with univariable and multivariable associations between sociodemographic factors and ever-self-testing for HIV in Malawi and Zimbabwe [file 12889_2020_8855_MOESM2_ESM.docx]

**Table S2. Univariable and multivariable associations between sociodemographic factors and ever-self-testing for HIV in Malawi and Zimbabwe, 2015-16**

| **Variables** | **Univariable (weighted)**  **n=24 683*** | | **Multivariable (weighted)**  **n=24 668*** | |
| --- | --- | --- | --- | --- |
|  | **Odds ratio** | **95% CI and p-value** | **Odds ratio** | **95% CI and p-value** |
| **Country** |  |  |  |  |
| Zimbabwe | 1 |  | 1 |  |
| Malawi | 0.74 | 0.53-1.04 | 0.81 | 0.53-1.24 |
| **Sex** |  |  |  |  |
| Female | 1 |  | 1 |  |
| Male | 1.27 | 0.93-1.74 | 1.05 | 0.75-1.46 |
| **Age** |  |  |  |  |
| **15-19** | 1 | p<0.002^§^ | 1 | p<0.017^§^ |
| 20-24 | 1.86 | 1.10-3.15 | 1.75 | 0.98-3.10 |
| 25-29 | 2.66 | 1.62-4.38 | 2.47 | 1.43-4.27 |
| 30-34 | 3.15 | 1.76-5.63 | 2.89 | 1.47-5.68 |
| 35-39 | 2.70 | 1.47-4.94 | 2.50 | 1.22-5.10 |
| 40-44 | 2.47 | 1.37-4.47 | 2.35 | 1.19-4.66 |
| 45+ | 1.56 | 0.82-3.00 | 1.41 | 0.69-2.91 |
| **Residence** |  |  |  |  |
| Urban | 1 |  | 1 |  |
| Rural | 0.36 | 0.25-0.50 | 0.76 | 0.45-1.27 |
| **HIV status** |  |  |  |  |
| HIV negative | 1 |  | 1 |  |
| HIV positive | 1.27 | 0.83-1.95 | 1.03 | 0.66-1.62 |
| **Marital status** |  |  |  |  |
| Single | 1 |  | 1 |  |
| Married or cohabiting | 1.23 | 0.88-1.73 | ** | ** |
| **Wealth** |  |  |  |  |
| Poorest | 1 | p<0.001^§^ | 1 | p<0.001^§^ |
| Poor | 1.82 | 0.94-3.52 | 1.81 | 0.94-3.49 |
| Middle | 1.21 | 0.59-2.49 | 1.22 | 0.60-2.50 |
| Rich | 2.11 | 1.12-3.96 | 1.74 | 0.90-3.38 |
| Richest | 4.79 | 2.59-8.85 | 3.59 | 1.79-7.18 |
| **Employment** |  |  |  |  |
| Not actively working | 1 |  | 1 |  |
| Actively working | 1.31 | 0.95-1.80 | 0.99 | 0.70-1.44 |
| **Education** |  |  |  |  |
| ≤Primary | 1 |  | 1 |  |
| ≥ Secondary | 3.08 | 2.20-4.32 | ** | ** |
| **Literacy** |  |  |  |  |
| Being illiterate | 1 |  | 1 |  |
| Being literate | 2.05 | 1.35-3.09 | 1.37 | 0.91-2.08 |
| **HIV-related risk***** |  |  |  |  |
| Low-risk | 1 | p<0.004^§^ | 1 | p<0.178^§^ |
| Moderate-risk | 1.35 | 0.96-1.93 | 1.06 | 0.74-1.52 |
| High-risk | 2.20 | 1.36-3.57 | 1.61 | 0.96-2.71 |

* Both samples were weighted based on standard DHS weights; Strata = 56; PSU=1 256. Not all participants were systematically surveyed on self-testing questions. Out of 31 385 participants, 24 683 were asked about HIV self-testing, resulting in smaller sample size. Among those reporting on HIV self-testing, 15 did not provide information on sexual activity and HIV risk. Population size asked about awareness or ever self-testing for HIV: 24 668 (HIV risk), 24 668 (sexual activity).

**Represents variables which were not included in the multivariable analysis due to identified collinearity.

*** HIV risk is defined in this analysis includes reported sexual activity in the past four weeks, and the following high-risk exposures in the previous 12 months: multiple (i.e. ≥2) partners, any paid sex (asked to men), having received gifts, cash or other compensation in exchange for sex (asked to women), and having a sexually transmitted infection (STI). Individuals with any “high-risk” exposures were classified as “high-risk”, with the remaining respondents classified as “low risk” if reporting no sexually activity in the past four weeks, and as “moderate risk” otherwise.

^§^ P-value based on Wald test. P-values for variables with more than two categories are shown.
