# Supplementary material for: Use and awareness of and willingness to self-test for HIV: an analysis of cross-sectional population-based surveys in Malawi and Zimbabwe
Source: BMC Public Health. 2020 May 25;20:779. doi: 10.1186/s12889-020-08855-7 (PMC7249304; doi:10.1186/s12889-020-08855-7)
Supplement: Supplementary file 3 — Additional File 3: Table S3. Univariable and multivariable associations between sociodemographic factors and willingness to self-test among men in Zimbabwe, by testing history, 2015–16. Supplementary data with univariable and multivariable associations between sociodemographic factors and willingness to self-test among men in Zimbabwe [file 12889_2020_8855_MOESM3_ESM.docx]

**Table S3. Univariable and multivariable associations between sociodemographic factors and willingness to self-test among men in Zimbabwe, by testing history, 2015-16**

| **Variables** | **Univariable (weighted)** | | | | **Multivariable (weighted)** | | | |
| --- | --- | --- | --- | --- | --- | --- | --- | --- |
|  | **Ever tested**  **(n=4 934)*** | | **Never tested**  **(n=2 437)*** | | **Ever tested**  **(n=4 934)*** | | **Never tested**  **(n=2 437)*** | |
|  | **OR** | **95% CI and p-value** | **OR** | **95% CI and p-value** | **aOR** | **95% CI and p-value** | **aOR** | **95% CI and p-value** |
| **Age** |  |  |  |  |  |  |  |  |
| **15-19** | **1** | p<0.001^§^ | **1** | p<0.001^§^ | **1** | p<0.004^§^ | **1** | p<0.003^§^ |
| 20-24 | 1.76 | 1.32-2-36 | 1.70 | 1.27-2.28 | 1.60 | 1.17-2.18 | 1.53 | 1.13-2.09 |
| 25-29 | 2.01 | 1.46-2.77 | 2.47 | 1.59-3.83 | 1.63 | 1.15-2.32 | 2.03 | 1.27-3.23 |
| 30-34 | 2.59 | 1.80-3.73 | 2.25 | 1.40-3.60 | 1.99 | 1.35-2.96 | 1.66 | 0.94-2.93 |
| 35-39 | 2.32 | 1.80-3.73 | 2.76 | 1.61-4.73 | 1.80 | 1.20-2.72 | 2.06 | 1.13-3.75 |
| 40-44 | 2.53 | 1.72-3.72 | 1.96 | 1.04-3.71 | 2.04 | 1.34-3.10 | 1.47 | 0.70-3.09 |
| 45+ | 2.08 | 1.49-2.92 | 1.27 | 0.85-1.87 | 1.71 | 1.18-2.48 | 0.93 | 0.58-1.51 |
| **Residence** |  |  |  |  |  |  |  |  |
| Urban | 1 |  | 1 |  | 1 |  | 1 |  |
| Rural | 1.05 | 0.83-1.34 | 0.95 | 0.73-1.24 | 1.08 | 0.73-1.60 | 0.99 | 0.61-1.62 |
| **Wealth** |  |  |  |  |  |  |  |  |
| Poorest | **1** | p<0.745^§^ | **1** | p<0.551^§^ | **1** | p<0.724^§^ | **1** | p<0.751^§^ |
| Poor | 1.20 | 0.83-1.74 | 1.16 | 0.83-1.64 | 1.22 | 0.84-1.76 | 1.20 | 0.92-1.55 |
| Middle | 1.00 | 0.71-1.42 | 0.94 | 0.69-1.27 | 1.06 | 0.74-1.51 | 1.02 | 0.75-1.40 |
| Rich | 1.11 | 0.74-1.66 | 1.21 | 0.84-1.76 | 1.09 | 0.72-1.63 | 1.17 | 0.80-1.72 |
| Richest | 0.99 | 0.70-1.39 | 0.96 | 0.67-1.37 | 0.92 | 0.56-1.50 | 0.97 | 0.55-1.73 |
| **Employment** |  |  |  |  |  |  |  |  |
| Not actively working | 1 |  | 1 |  | 1 |  | 1 |  |
| Actively working | 1.51 | 1.24-1.83 | 1.58 | 1.29-1.94 | 1.14 | 0.91-1.42 | 1.20 | 0.94-1.53 |
| **Education** |  |  |  |  |  |  |  |  |
| ≤Primary |  |  |  |  | 1 |  | 1 |  |
| ≥ Secondary | 1.28 | 1.00-1.64 | 1.16 | 0.95-1.42 | ** | ** | ** | ** |
| **Literacy** |  |  |  |  |  |  |  |  |
| Being illiterate | 1 |  | 1 |  | 1 |  | 1 |  |
| Being literate | 1.39 | 1.06-1.83 | 1.17 | 0.91-1.50 | 1.42 | 1.06-1.90 | 1.19 | 0.92-1.55 |
| **HIV status** |  |  |  |  |  |  |  |  |
| HIV negative | 1 |  | 1 |  | 1 |  | 1 |  |
| HIV positive | 0.93 | 0.64-1.35 | 2.26 | 1.29-3.97 | 0.79 | 0.55-1.14 | 1.83 | 1.03-3.24 |
| **Marital status** |  |  |  |  |  |  |  |  |
| Single | 1 |  | 1 |  | 1 |  | 1 |  |
| Married or cohabiting | 1.84 | 1.48-2.29 | 1.61 | 1.25-2.07 | ** | ** | ** | ** |
| **HIV-related risk***** |  |  |  |  |  |  |  |  |
| Low-risk | 1 | p<0.001^§^ | 1 | p<0.001^§^ | 1 | p<0.505^§^ | 1 | p<0.250^§^ |
| Moderate-risk | 1.91 | 1.52-2.41 | 1.66 | 1.26-2.18 | 1.40 | 1.07-1.82 | 1.17 | 0.81-1.79 |
| High-risk | 1.58 | 1.18-2.11 | 1.97 | 1.31-2.95 | 1.42 | 0.90-1.67 | 1.39 | 0.93-2.17 |
| **Aware self-test** |  |  |  |  |  |  |  |  |
| No | 1 |  | 1 |  | 1 |  | 1 |  |
| Yes | 1.17 | 0.87-1.57 | 0.96 | 0.66-1.41 | 1.11 | 0.82-1.52 | 0.79 | 0.56-1.18 |

******Weighted analysis using standard DHS sample weights; Strata = 19; PSU=400. Out of 7 420 men surveyed, 7 372 reported on willingness to self-test. 48 respondents did not respond to this question on willingness. Because 1 person did not provide information on sexual activity and sexual risk resulting in sample size 7 321 for both HIV risk.

**Represents variables which were not included in the multivariable analysis due to identified collinearity.

*******HIV risk is defined in this analysis includes reported sexual activity in the past four weeks, and the following high-risk exposures in the previous 12 months: multiple (i.e. ≥2) partners, any paid sex (asked to men), having received gifts, cash or other compensation in exchange for sex (asked to women), and having a sexually transmitted infection (STI). Individuals with any “high-risk” exposures were classified as “high-risk”, with the remaining respondents classified as “low risk” if reporting no sexually activity in the past four weeks, and as “moderate risk” otherwise.

^§^ P-value based on Wald test. P-values for variables with more than two categories are shown.
